# Bioprospecting of Ureolytic Bacteria From Laguna Salada for Biomineralization Applications

**DOI:** 10.3389/fbioe.2018.00209

**Published:** 2019-01-18

**Authors:** Dayana Arias, Luis A. Cisternas, Carol Miranda, Mariella Rivas

**Affiliations:** ^1^Departamento de Ingeniería Química y Procesos de Minerales, Universidad de Antofagasta, Antofagasta, Chile; ^2^Laboratorio de Biotecnología Algal y Sustentabilidad (BIOAL), Departamento de Biotecnología, Facultad de Ciencias del Mar y Recursos Biológicos, Universidad de Antofagasta, Antofagasta, Chile; ^3^Centro de Investigación Científico Tecnológico Para la Minería, Antofagasta, Chile

**Keywords:** biomineralization, ureolytic bacteria, struvite, monohydrocalcite, bioprospecting, urease

## Abstract

The processes of biomineralization, mediated by ureolytic bacteria, possess a wide range of technological applications, such as the formation of biocements and remediation of water and soil environments. For this reason, the bioprospecting of new ureolytic bacteria is interesting for its application to these technologies, particularly for water treatment. This study demonstrates the isolation, selection, and identification of halotolerant ureolytic bacteria from Laguna Salada (inland from Atacama Desert) and the evaluation of their ability to precipitate calcium carbonate crystals in freshwater in the presence of calcium ions, as well as the ability to induce the precipitation of crystals from different ions present in seawater. Twenty-four halotolerant ureolytic bacteria whose molecular identification gives between 99 and 100% identity with species of the genus *Bacillus, Porphyrobacter, Pseudomonas, Salinivibrio*, and *Halomonas* were isolated. When cultivated in freshwater, urea, and calcium chloride, all species are able to biomineralize calcium carbonate in different concentrations. In seawater, the strains that biomineralize the highest concentration of calcium carbonate correspond to *Bacillus subtilis* and *Halomonas* sp. SEM–EDX and XRD analyses determined that both bacteria induce the formation of 9–33% halite (NaCl), 31–66% monohydrocalcite (CaCO_3_ × H_2_O), and 24–27% struvite (MgNH_4_PO_4_ × 6H_2_O). Additionally, *B.subtilis* induces the formation of 7% anhydrite (CaSO_4_). In seawater, *B.subtilis* and *Halomonas* sp. were able to precipitate both calcium (96–97%) and magnesium (63–67%) ions over 14 days of testing. Ion removal assays with *B.subtilis* immobilized in beads indicate a direct relationship between the urea concentration and a greater removal of ions with similar rates to free cells. These results demonstrate that the biomineralization mediated by bacterial urea hydrolysis is feasible in both freshwater and seawater, and we propose its application as a new technology in improving water quality for industrial uses.

## Highlights

- It is proposed to use ureolytic bacteria for the selective removal of calcium and magnesium from seawater.
- *Bacillus subtilis* produces the highest concentration of monohydrocalcite, close to 7,802 mg/L.
- In biomineralization tests, *B.subtilis* and *Halomonas* sp. removes 97 and 96% of the calcium ion in 5 days and 67 and 63% of magnesium in 14 days, respectively.
- The XRD analysis determined that the crystals formed by *B.subtilis* strain LN8B correspond to 33% monohydrocalcite, 33% halite, 27% struvite, and 7% anhydrite.

## Introduction

Biomineralization refers to the process in which living organisms form minerals (Dove et al., 2004; Achal et al., 2015). There are several types of biominerals, such as organic crystals, oxides, hydroxides, sulfates, sulfides, chlorides, phosphates, and carbonates (Barabesi et al., 2007; Achal et al., 2009; Silva-Castro et al., 2015). Each biomineral has specific properties, like an unique size, crystal structure, and isotopic composition of trace elements (Perry et al., 2007; Cartwright et al., 2012). From all the biominerals known so far, carbonate precipitation, specifically of calcium carbonates, has become greatly important due to its wide range of technological applications, such as the formation of biocements, remediation of water and soil environments, and uptake of carbon dioxide (Hammes et al., 2003; Dhami et al., 2013; Phillips et al., 2013; Achal et al., 2015).

Microorganisms possessing specific metabolisms, including denitrifying, iron-reducing, sulfate-reducing, and ureolytic bacteria, play key roles during carbonate biomineralization (DeJong et al., 2010; Dhami et al., 2013). Particularly, ureolytic bacteria create an increase in ambient pH, favoring the formation of supersaturated conditions necessary for carbonate precipitation (Whiffin et al., 2007; DeJong et al., 2010). Ureolytic bacteria use the action of urease (urea amidohydrolase; EC 3.5.1.5), which catalyzes urea hydrolysis to produce ammonium and carbamate. The carbamate spontaneously decomposes to produce an ammonium and carbonic acid molecule. Both ammonium molecules and the carbonic acid in aqueous solution, in equilibrium with their deprotonated and protonated forms, generate an increase in the pH of the solution, which induces ion precipitation (Al-Thawadi, 2011; Cheng and Cord-Ruwisch, 2013; Anbu et al., 2016).

An attractive application of biomineralization mediated by ureolytic bacteria is the pretreatment of water (Arias et al., 2017a). Industrial wastewaters, hard water, and groundwater have excessive levels of contaminants, such as heavy metals, radionuclides, phosphates, and salts. On the other hand, seawater is characterized by a high SO_4_/HCO_3_ molar ratio, generally between 11 and 15 (El-Manharawy and Hafez, 2003), with an abundance of calcium sulfate (>90% wt/wt) over other species, such as CaCO_3_, MgSO_4_, MgCO_3_, MgSiO_3_, SiO_2_, and CaPO_4_ (Millero et al., 2008). Specifically, calcium and magnesium ions, in the presence of SO${}_{4}^{2-}$, play an important role in calcium carbonate scaling (Waly et al., 2012; Li et al., 2015). Furthermore, the Cl^−^ ion can react with the Fe^2+^ ions that are present to create FeCl_2_. This reacts with dissolved oxygen, generating Fe_2_O_3_ and FeCl_3_, which are strong oxidizing agents of pipes and other equipment (Liang et al., 2013).

The advantages of using this type of microorganism is their ability to efficiently immobilize toxic metals, radionuclides, or ions by precipitation or coprecipitation with CaCO_3_, dependent on metal valence status and redox potential (Kumari et al., 2016).

Numerous studies have been carried out using *Sporosarcina pasteurii* in processes of biomineralization and coprecipitation of CaCO_3_ because it is non-pathogenic and has high urease activity (Whiffin et al., 2007; Achal et al., 2009; Al-Thawadi, 2011). However, studies on alternative species for the hydrolysis of urea are scarce, especially of ecologically relevant species capable of surviving a given remediation site. For these reasons, the search for biotechnologically relevant microbes capable of removing the previously described minerals/elements is of great interest. Here, the bioprospecting of halotolerant ureolytic bacteria from Laguna Salada, the evaluation of the bacterial capacity to precipitate calcium carbonate crystals in freshwater in the presence of calcium ions, and the ability to induce the precipitation of crystals from the different ions present in seawater are evaluated.

This evaluation is our first approximation for using biomineralization processes to pretreat freshwater and seawater to improve the quality for industrial processes, such as pretreatment for reverse osmosis desalination and mineral processing.

## Materials and Methods

### Study Area

The sampling site is located in the water system from Peine village on the south edge of the Atacama Salar, which is formed by three superficially connected lagoons: Salada, Saladita, and Interna. Samples of water and sediments from sites LS1, LS2, and LS3 were collected in June 2014 from Laguna Salada, located at 2,300 meters above sea level (m.a.s.l.) at latitude 23°S and at longitude 68°W in the Chilean Altiplano. The geographical location, altitude, and physicochemical parameters of the points sampled in this site are shown in Table 1.

**Table 1:** Physicochemical parameters of sampling sites at Laguna Salada.

**Sites**	**Latitude S**	**Longitude W**	**Altitude (m.a.s.l.)**	**Water temperature ^**°**^C**	**pH**	**O_**2**_ (mg/L)**	**NaCl (mg/L)**	**Ca (mg/L)**	**Mg (mg/L)**
LS1	23° 39′ 5′	68° 10′ 0″	2293	14.70	7.56	5.44	274,000	2760	4880
LS2	23° 39′12′	68° 8′ 24″	2282	25.00	7.98	6.41	274,000	3240	800
LS3	23° 39′12′	68° 8′ 19″	2293	13.20	8.13	16.52	93,000	2320	1600

### Bioprospecting of Ureolytic Bacteria

Environmental samples were taken in sterile plastic tubes and stored at −20°C for further analysis. Five grams of samples were enriched in 50 mL of the following four culture media: TN medium (Tryptone Soya Broth (TSB, DIFCO TM) dissolved in freshwater and supplemented with 35 g/L NaCl), TM medium (TSB dissolved in seawater), LN culture medium (LB (MoBio Lab., Inc.) dissolved in freshwater and supplemented with 35 g/L NaCl), and LM medium (LB dissolved in seawater). For the isolation of cultured halophilic bacteria, the described culture media were prepared on plates with 15 g/L agar (Oxoid); 10 μL of each enriched sample was added and incubated at 30°C until growth was observed. The colony forming units (CFU) were selected according to their morphology and sub-cultured to identify the presence of urease activity.

In order to determine the presence of urease activity, the bacterial isolates were cultured in tubes with Christensen's Urea Agar, modified with 20 g/L NaCl (Atlas, 2010; Arias et al., 2017b). The bacterial isolates were seeded on the agar surface and incubated at 37°C until a coloration change from yellow to pink was observed. Trials were performed in triplicate. *Proteus mirabilis* was used as a positive control and *Escherichia coli* JM109 as a negative control.

### Identification of Microbial Strains

Genomic DNA was extracted using the Power Soil kit (Mobio Cat. No. 1288-100), according to the manufacturer's instructions. PCR amplification of the rRNA gene was performed using universal primers 27F (5′-AGAGTTTGATCMTGGCTCAG-3′) and 1492R (5′TACGGYTACCTTGTTACGACTT-3′) (Frank et al., 2008). The samples were sequenced at Macrogen Inc. (South Korea). The sequences were edited using FinchTV software version 1.4 (www.geospiza.com). The sequences were then assembled using ChromasPro version 1.6 (2012) and analyzed using the BLASTN database (www.ncbi.nlm.nih.gov) using the non-redundant GenBank nucleotide collection. The identified bacterial strain sequences were then deposited in GenBank (KX185693, KX185689, KX185695, KX185692, KX185696, KX185685, KX185686, KX185691, KX185687, KX185688, KX185690, KX185684, KX018264, KX018260, KX018261, KX018262, KX018263, KX018265, KX018266, KX018267, KX018268, KX018269, KX018270, KX185694, and KX018271).

### Analysis of Nucleotide Sequence and Access Numbers

**Figure 1:**
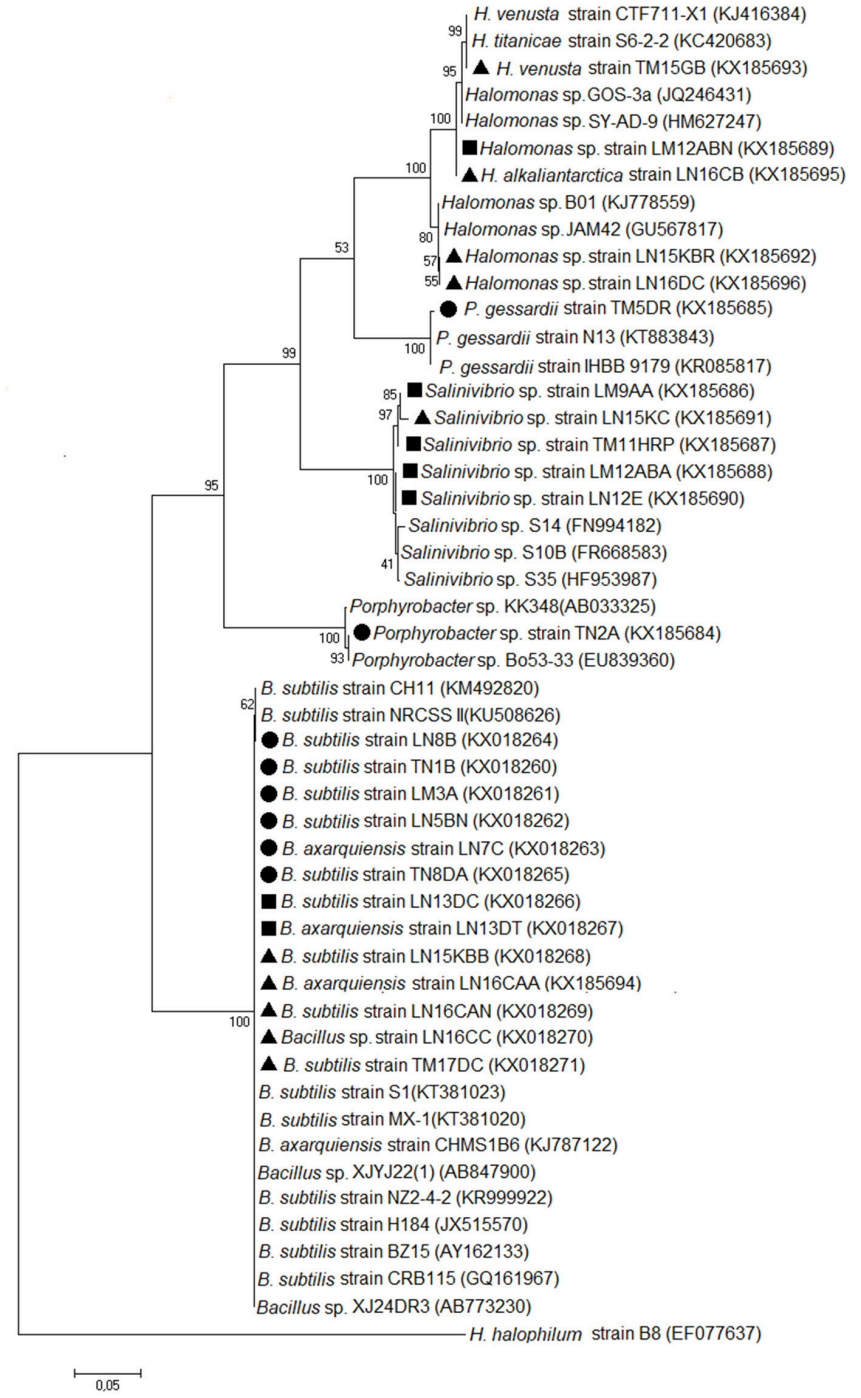
Molecular phylogenetic analysis by Maximum Likelihood method. The evolutionary history was inferred by using the Maximum Likelihood method based on the General Time Reversible model (Nei and Sudhir, 2000). The tree with the highest log likelihood (−6,288, 2,707) is shown. The percentage of trees in which the associated taxa clustered together is shown next to the branches. Initial tree(s) for the heuristic search were obtained automatically by applying Neighbor-Joining and BioNJ algorithms to a matrix of pairwise distances estimated using the Maximum Composite Likelihood (MCL) approach and by selecting the topology with a superior log likelihood value. The tree is drawn to scale, with branch lengths measured in the number of substitutions per site. The analysis involved 50 nucleotide sequences. Codon positions included were 1st + 2nd + 3rd + Noncoding. All positions containing gaps and missing data were eliminated. There were a total of 1,268 positions in the final dataset. Evolutionary analyses were conducted in MEGA7 (Kumar et al., 2016). The circle corresponds to LS1. The square corresponds to LS2. The triangle corresponds to LS3.

Evolutionary analyses were conducted in MEGA7.0 using Maximum Likelihood with a bootstrap of 1,000. The model used was General Time Reversible, while the branch swap filter analysis setting used was weak. The tree is drawn to scale, with branch lengths measured in the number of substitutions per site. The nucleotide sequences from this study are available in GenBank (http://www.ncbi.nlm.nih.gov) under access numbers indicated in brackets at the tree in Figure 1.

### Analysis of Biomineralization Capacity in Water

The strains were grown first in LN medium, with 20 g/L of CaCl_2_ × 2H_2_O, and later in LM medium, both with 20 g/L urea. The assays were called the freshwater and seawater biomineralization test, respectively. All assays were performed in triplicate and incubated for 7 days at 30°C and 120 rpm. The pH of the assays was measured daily. At the end of day 7, the concentrations of total carbonates, bicarbonates, calcium, and soluble magnesium were quantified. The medium without bacteria acted as the negative control. The precipitates obtained were washed three times with distilled water and dried for 24 h at 60°C for SEM–EDX and XRD analyses.

### Biomass Immobilization in Polyvinyl Alcohol-Alginate Beads

Concentrated cells of *B.subtilis* strain LN8B were mixed with a 12 and 2% of polyvinyl alcohol solution (Sigma-Aldrich, MW 89,000–98,000, 99% hydrolyzed) and alginic acid (Sigma-Aldrich), respectively, to a final concentration of 4.5 × 10^9^ cells/ml. The mixture was dripped via a peristaltic pump into a sterile solution of 4% CaCl_2_ × H_2_O (Merck) and 4% H_3_BO_3_ (Merck) for 2 h to form immobilized cell spheres of 0.4 cm diameter. After 2 h, the beads were washed 3 times with sterile distilled water and stored at 4°C until use.

### Experiments of Ca^2+^ and Mg^2+^ Biomineralization From Seawater (SW) With Immobilized Biomass

The removal studies with immobilized biomass of *B.subtilis* strain LN8B were performed in 250 mL Erlenmeyer flasks at an incubation temperature of 30°C and constant shaking at 120 rpm for 2 weeks. The concentrations of urea evaluated were 5, 10, 20, and 30 g/L, and the ratio of SW:spheres were of 1:1.

### Chemical Analysis

Calcium and magnesium ions were analyzed by atomic absorption spectrophotometry (220FS, Varian), with direct suction. Total carbonates and bicarbonates were measured through acid–base titration (Jagadeesha Kumar et al., 2013).

### Ammonium Quantification

As a measure of urease activity, the concentration of ammonium produced from urea hydrolysis was quantified according to the phenol-hypochlorite method (Achal et al., 2009).

### SEM–EDX Analyses

Crystal morphology visualization was done using a scanning electron microscope (JEOL model JSM 6360LV) coupled to an X-ray detector (Oxford Inca). The samples were prepared on a carbon tape and covered with gold via sputtering (Denton Desk II). The secondary electron current used was between 10 and 20 kV. The magnification was between 250 × and 5,000 ×. The working distance used was 10 nm, with a spot size between 40 and 55.

### XRD Analyses

The XRD analyses were obtained using a diffractometer (Siemens D5000) with a graphite secondary monochromator and CuKα radiation. Data were collected for a 1.0 s integration time in 0.02° 2θ steps at 40 kV and 30 mA. Scanning was from 3° to 70° 2θ. The components of the sample were identified by comparing them with standards established by the International Center for Diffraction Data.

### Statistical Analysis

Unless otherwise indicated, the presented data are the means of three independent experiments. The standard deviations of each set of experiments are represented in the corresponding figure (as bars). The comparisons of the mean CaCO_3_ production between isolated bacteria were conducted to identify statistical significance. This was done using Minitab software version 17.0 (www.minitab.com) through unidirectional ANOVA and Dunnett tests or Student's *t*-test and Tukey test a posteriori. In all cases, comparisons for which *p* < 0.05 were considered significant.

## Results

### Bioprospecting of Ureolytic Bacteria

The enrichment of each sample in 4 different media generated a total of 72 enrichment cultures, resulting in 104 colony forming units (CFU) with different appearances, colorations, and morphologies. Twenty-four bacterial isolates demonstrated the ability to hydrolyze urea in the Christensen's Urea Agar assay (23.07% of total isolates). All isolates with ureolytic activity were phylogenetically identified based on their 16S rRNA sequences by BLASTN. 98–100% identity was obtained with species of the *Bacillus, Salinivibrio, Halomonas, Pseudomonas*, and *Porphyrobacter* genera (Table 2).

**Table 2:** Phylogenetic identification of halotolerant ureolytic bacterial strains isolated from Laguna Salada.

**Bacterial strain**	**Closest species in BLASTN^a^**	**E value**	**Coverage (%)**	**Identity (%)**	**Homolog GenBank accession no**.
TN1B	*Bacillus subtilis* S1	0.0	100	100	KT381023
TN2A	*Porphyrobacter* sp.	0.0	100	99	EU839360
TN8DA	*Bacillus subtilis* NZ2-4-2	0.0	100	100	KR999922
TM5DR	*Pseudomonas gessardii* N13	0.0	100	99	KT883843
TM11HRP	*Salinivibrio* sp. S10B	0.0	100	98	FR668583
TM15GB	*Halomonas venusta* CTF711-X1	0.0	100	99	KJ416384
TM17DC	*Bacillus subtilis* NZ2-4-2	0.0	100	100	KR999922
LM3A	*Bacillus subtilis* S1	0.0	100	100	KT381023
LM9AA	*Salinivibrio* sp. S10B	0.0	100	99	FR668583
LM12ABA	*Salinivibrio* sp. S10B	0.0	100	99	FR668583
LM12ABN	*Halomonas* sp. GOS-3a	0.0	100	99	JQ246431
LN5BN	*Bacillus subtilis* S1	0.0	100	100	KT381023
LN7C	*Bacillus axarquiensis* CHMS1B6	0.0	100	99	KJ787122
LN8B	*Bacillus subtilis* CH11 16S	0.0	100	99	KM492820
LN12E	*Salinivibrio* sp. S10B	0.0	100	99	FR668583
LN13DC	*Bacillus subtilis* H184 16S	0.0	100	100	JX515570
LN15KBB	*Bacillus subtilis* S1	0.0	100	99	KT381023
LN15KBR	*Halomonas* sp. JAM42	0.0	99	99	GU567817
LN15KC	*Salinivibrio* sp. S10B	0.0	100	99	FR668583
LN16CAA	*Bacillus axarquiensis* CHMS1B6	0.0	100	99	KJ787122
LN16CAN	*Bacillus subtilis* CRB115	0.0	100	99	GQ161967
LN16CB	*Halomonas alkaliantarctica* CRSS	0.0	100	99	NR114902
LN16CC	*Bacillus* sp. XJ24DR3	0.0	100	99	AB773230
LN16DC	*Halomonas* sp. JAM42	0.0	100	99	GU567817

a *Sequence homology was determined with BLASTN*.

From the isolates with urease activity, 50% are of the *Bacillus* species, 20.83% are of the *Salinivibrio* species, and 20% are of the *Halomonas* species. The TN2A isolate, making up 4.17% of the isolates, is closely related to the *Porphyrobacter* species, and the TM5DR strain, making up another 4.17%, is *Pseudomonas gessardii*. These last two had 99% identity. A phylogenetic tree was generated based on the 16S rRNA. The relatedness of the sequences is shown in Figure 1.

### Analysis of Biomineralization Capacity in Water

**Figure 2:**
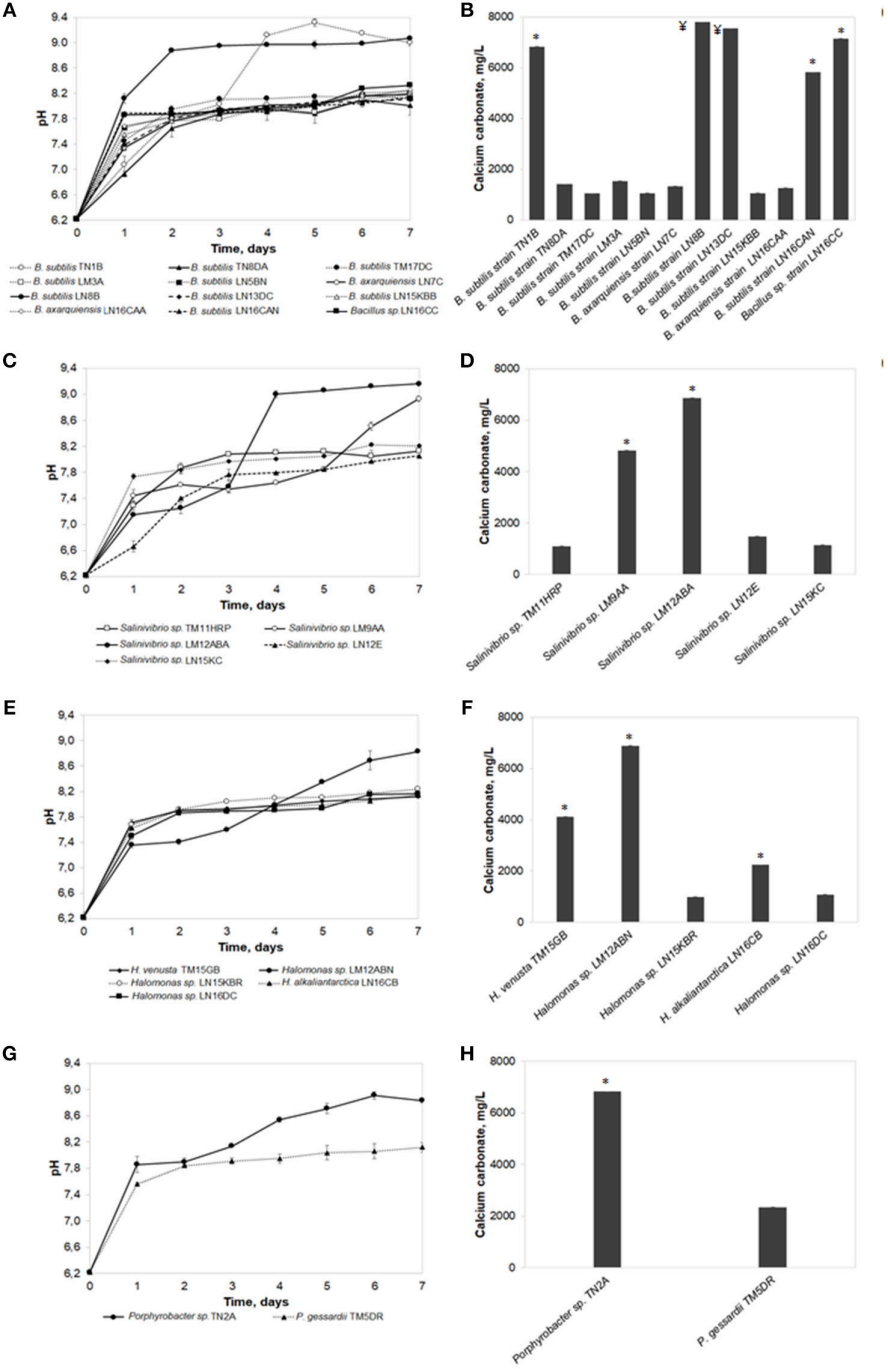
The freshwater biomineralization test. pH evolution and calcium carbonate precipitation of bacterial strains belonging to *Bacillus* genus **(A,B)**, *Salinivibrio* genus **(C,D)**, *Halomonas* genus **(E,F)**, and others genera **(G,H)**. Error bars show the standard deviations for three replications per sample. Asterisks indicate significant differences (*p* < 0.05) among the values. ¥ indicates no significant differences among the values but there being differences with respect to all the other isolates.

The freshwater biomineralization test was performed with all the isolates, evaluating the total produced carbonates and the pH evolution at the end of the seventh day (Figure 2). All identified strains were capable of forming calcium carbonate crystals. Quantification of the produced carbonates and pH was grouped by bacterial genus. Results showed that the highest concentration in the *Bacillus* genus was produced by *Bacillus subtilis* strain LN8B (7,802 ± 0.025 mg/L; pH of 9.07 ± 0.03). This was followed, in descending order, by *B.subtilis* strain LN13DC (7,547 ± 0.025 mg/L; pH of 8.25 ± 0.03), *Bacillus* sp. strain LN16CC (7,153 ± 0.087 mg/L; pH of 8.33 ± 0.08), and *B.subtilis* TN1B (6,834 ± 0.049 mg/L; pH of 9.00 ± 0.04). In the other genera, highlights include *Halomonas* sp. strain LM12ABN (6,881 ± 0.076 mg/L; pH of 8.83 ± 0.07), *Salinivibrio* sp. strain LM12ABA (6,871 ± 0.048 mg/L; pH of 9.16 ± 0.04), *B.subtilis* strain, and *Porphyrobacter* sp. strain TN2A (6,830 ± 0.019 mg/L; pH of 8.84 ± 0.03). No biomineralization signals or precipitate formations were observed in the controls without bacteria.

Regarding the pH evolution on the seventh day of the bioassay, in most cultures, a direct relationship was observed between higher concentrations of total precipitated carbonate and higher final pH levels reached by *B.subtilis* strain LN8B. The pH of the negative control remained constant throughout the test at 6.34 ± 0.01.

**Figure 3:**
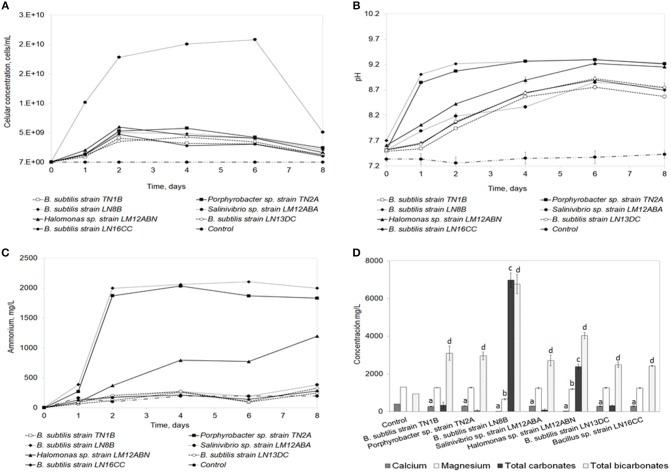
Seawater biomineralization test. Analysis of the ammonium production **(A)**, pH evolution **(B)**, cellular density **(C)**, and ion quantification **(D)**. Error bars show the standard deviations for three replications per sample. “a” indicates significant differences with respect to control for calcium. “b” indicates significant differences with respect to the control for magnesium. “c” indicates significant differences with respect to the control for total carbonates. “d” indicates significant differences with respect to the control for total bicarbonates.

The strains that precipitated the highest concentration of calcium carbonate (>6 g/L) were selected to perform the seawater biomineralization test (Figure 3). Of the strains evaluated in these conditions, *Salinivibrio* sp. strain LM12ABA grew to the highest cell density (Figure 3A) (2 × 10^10^ cells/mL). The pH of the medium increased over time for all strains, with *B*. *subtilis* LN8B (Figure 3B), *Porphyrobacter* sp. strain TN2A, and *Halomonas* sp. strain LM12ABN showing the highest pH change relative to the control (pH of 7.35 ± 0.10). When evaluating ammonium production as an indirect measure of urease activity, the same trend was observed in these three strains (Figure 3C).

The quantification of total calcium, magnesium, carbonates, and bicarbonates of the seven strains, compared to the control, in the seawater biomineralization test are shown in Figure 3D. The greatest ion biomineralization compared to the control was from calcium (concentration of Ca^2+^ in the control was 403.33 ± 2.31 mg/L). *B.subtilis* strain TN1B, *Porphyrobacter* sp. strain TN2A, *Salinivibrio* sp. strain LM12ABA, *B.subtilis* strain LN13DC, and *Bacillus* sp. strain LN16CC biomineralize Ca^2+^, decreasing its concentration, on average, by 27.92% after 7 days of bioassay. *B.subtilis* strain LN8B showed decreases of 98%, and *Halomonas* sp. strain LM12ABN showed decreases of 92.77%. Magnesium biomineralization—evidenced by decreases in the concentration of Mg^2+^ in solution in the presence of *B.subtilis* strain TN1B, *Porphyrobacter* sp. strain TN2A, *Salinivibrio* sp. strain LM12ABA, *B.subtilis* strain LN13DC, and *Bacillus* sp. strain LN16CC—was only reduced 2.75% compared to the control (concentration of Mg^2+^ in control was 1300.67±2.31 mg/L). In contrast, the *B.subtilis* strain LN8B diminished Mg^2+^ concentrations by 49.32% compared to the control, reaching a final concentration of 659.2 ± 37.5 mg/L, while *Halomonas* sp. strain LM12ABN decreased Mg^2+^ concentrations by 7.86%. Concentrations of total carbonates and bicarbonates at the end of the bioassay generated by *B.subtilis* strain LN8B were 9773.1 ± 386.7 mg/L and 6767.2 ± 512.7 mg/L, respectively. In comparison, *Halomonas* sp. strain LM12ABN produced 2387.22 ± 130.69 and 4030.12 ± 164.37, respectively (Figure 3D).

**Figure 4:**
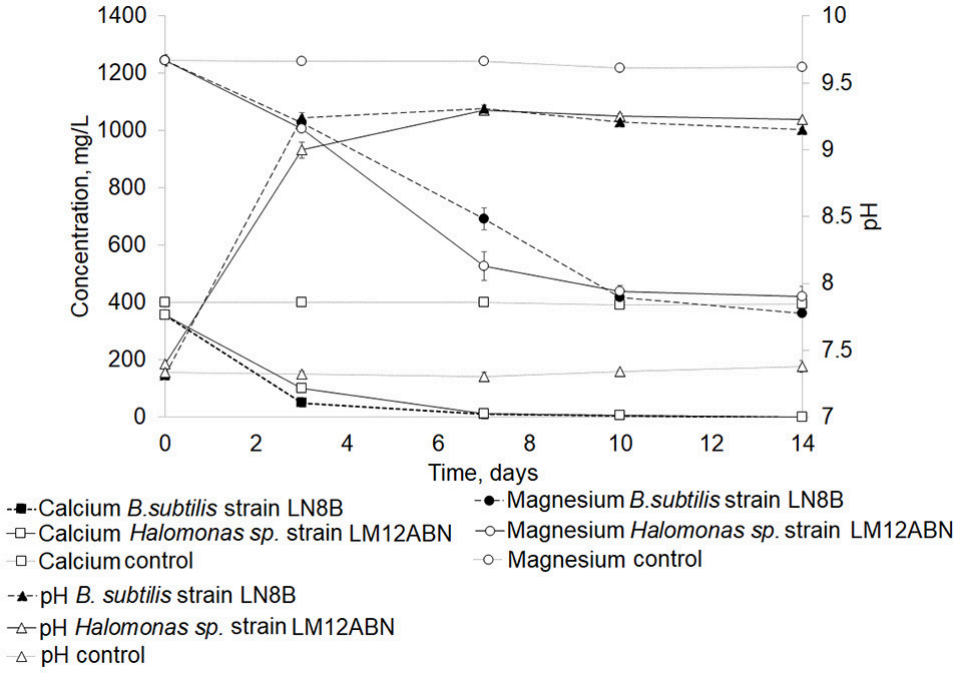
Evolution of the biomineralization of calcium and magnesium ions in seawater by *B. subtilis* strain LN8B and *Halomonas sp*. strain LM12ABN at day 14 of the cultures. Error bars show the standard deviations for three replications per sample.

Since *B.subtilis* strain LN8B and *Halomonas* sp. strain LM12ABN were the strains capable of biomineralizing the greatest amount of calcium and magnesium ions and producing the highest concentrations of total carbonates and bicarbonates. These strains were subjected to a new seawater biomineralization test for 14 days to determine the evolution of ion biomineralization. On the fifth day of the bioassay, *B.subtilis* strain LN8B, and *Halomonas* sp. strain LM12ABN biomineralized 97 and 96% of the calcium, respectively (Figure 4). Concerning the biomineralization of magnesium, by the third day of the bioassay, 67 and 63% of Mg^2+^ was biomineralized by *B.subtilis* strain LN8B and *Halomonas* sp. strain LM12ABN, respectively. For both bacteria, a significant increase in pH also happened on the third day of the bioassay, rising from 7.5 to 9.3 (Figure 4).

### SEM–EDX and XRD Analysis

**Figure 5:**
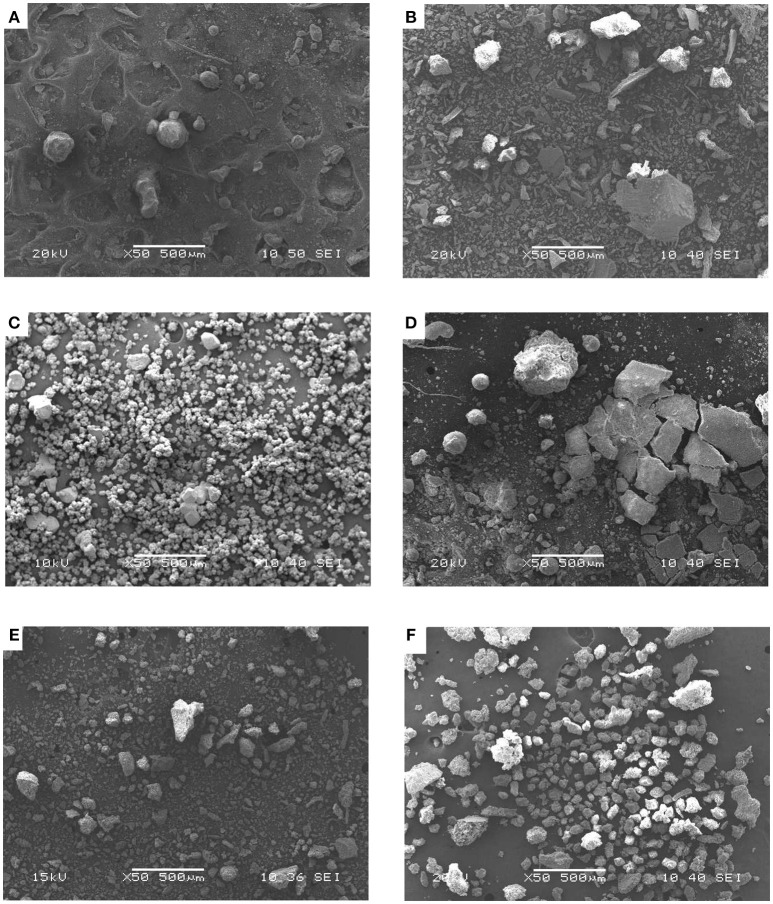
Scanning electron micrograph of crystals precipitated in freshwater by *B.subtilis* strain LM3A **(A)**, *B. axarquiensis* strain LN7C **(B)**, *B.subtilis* strain LN8B **(C)**, *Salinivibrio sp*. strain LM9AA **(D)**, *Halomonas sp*. strain LM12ABN **(E)**, and *P. gessardii* strain TM5DR **(F)**.

The scanning electron micrographs of the precipitates produced by some of the strains in the freshwater biomineralization assays are shown in Figure 5.

**Figure 6:**
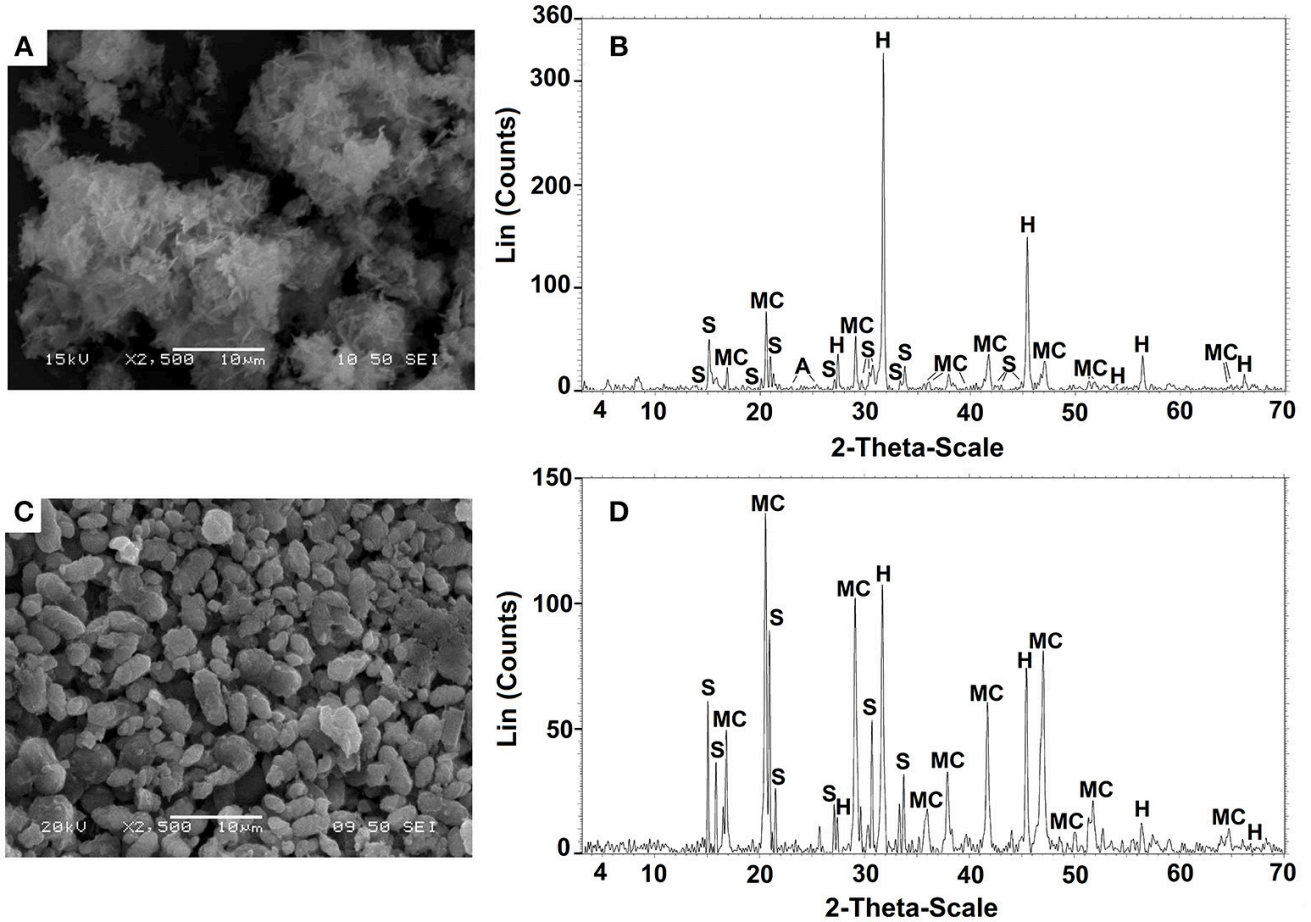
SEM and XRD analysis of the precipitates produced by *B.subtilis* strain LN8B **(A,B)** and *Halomonas* sp. strain LM12AB **(C,D)**. Struvite, monohydrocalcite, and halite were represented by (S), (M), and (H), respectively.

**Figure 7:**
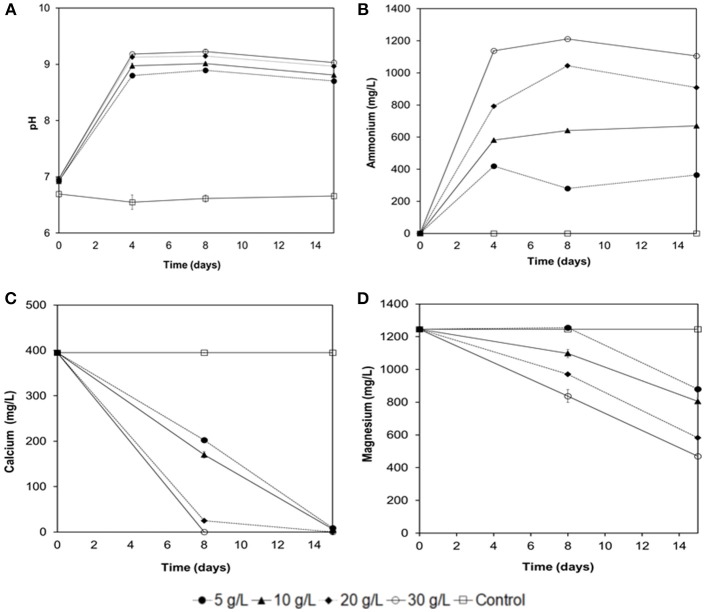
Experiments of Ca^2+^ and Mg^2+^ removal from seawater with immobilized biomass of *B.subtilis* strain LN8B at different concentrations of urea. Evolution of the pH **(A)**, ammonium **(B)**, calcium **(C)**, and magnesium **(D)** ions at the 15th day of the assay. Error bars show the standard deviations for three replications per sample.

The precipitates biomineralized by *B.subtilis* strain LN8B and *Halomonas* sp. strain LM12ABN in the seawater biomineralization test at the end of the bioassay are shown in Figures 6A,C, respectively. EDX analyses indicated the presence of C, Ca^2+^, Na^+^, Cl^−^, and Mg^+^ (data not shown). These results were corroborated by XRD (Figures 6B, 7D), indicating that *B.subtilis* strain LN8B is capable of inducing the precipitation of 33.08% halite (NaCl), 31.35% monohydrocalcite (CaCO_3_ × H_2_O), 27.73% struvite (MgNH_4_PO_4_ × 6H_2_O), and 7.84% anhydrite (CaSO_4_) (Figure 6B). The composition of the biominerals generated by *Halomonas* sp. strain LM12ABN was 9.40% halite, 66.03% monohydrocalcite, and 24.57% struvite (Figure 6D).

### Ca^2+^ and Mg^2+^ Removal Capacity From SW at Different Urea Concentrations With Immobilized Biomass of *B. Subtilis* Strain LN8B

Figure 7 shows the first approximation of the influence of urea concentration on the removal of Ca^2+^ and Mg^2+^ from SW with immobilized biomass in a PVA-Al matrix of *B. sutilis* strain LN8B. It is observed that the urea concentration has no great influence on the pH increase in SW (Figure 7A). The increase in pH as the urea concentration increases does not exceed 9.3 because of the equilibrium of the ammonium buffer. However, under the conditions tested, there is a direct relationship between urea concentration, ammonium production, and removal of the ions of interest (Figures 7B–D). It is also evidenced, as with free cells, that there is a tendency to precipitate Ca^2+^ ions first, then Mg^2+^ ions. This trend is also marked because the latter ion has a higher concentration in SW. Finally, the removal of Ca^2+^ with the immobilized cells reached 100 and 94% for urea concentrations of 20 and 30 g/L, respectively, at 8 days of testing. Removal of Mg^2+^ reaches 53 and 62% for the same concentrations of urea at 15 days of testing. These values are similar to those observed with free cells and can be optimized, for example, by increasing the cell concentration in the PVA-Al beads.

## Discussion

### Bacterial Biodiversity With Urease Activity From Laguna Salada

The biomineralization process through bacterial urea hydrolysis is one of the studied mechanisms that has a great potential to produce various biomaterials at high concentrations in short periods of time (Bachmeier et al., 2002; Al-Thawadi, 2011). Particularly, urease activity is widely distributed in organisms present in the water and soil (Burbank et al., 2012). Lloyd and Sheaffe (1973) estimated that only 17–30% of aerophilic, microaerophilic, and anaerobic microorganisms are capable of hydrolyzing urea. However, various studies have identified ureolythic bacteria in different environments, including *Acinobacter* sp. (Sanchez-Moral et al., 2003), *Deleyahlophila* (Rivadeneyra et al., 1996), *E. coli* (Bachmeier et al., 2002), and *Myxococcus xanthus* (Holt et al., 1993; González-Muñoz et al., 2000). In our study we demonstrate the selection of halotolerant bacteria with ureolytic activity from Laguna Salada in the Atacama Desert. Specifically, 24 bacterial isolates demonstrated the ability to hydrolyze urea in the Christensen's Urea Agar assay (23.07% of total isolates). All isolates with ureolytic activity were phylogenetically identified based on their 16S rRNA sequences by BLASTN. 98–100% identity was obtained with species from the *Bacillus, Salinivibrio, Halomonas, Pseudomonas*, and *Porphyrobacter* genera (Table 2).

*Bacillus* bacteria are widely distributed in natural environments and have been widely described for their ability to precipitate calcium carbonate (Castanier et al., 2000; Hammes et al., 2003), which coincides with 50% of the isolates in this study. Its easy cultivation and ability to absorb heavy metals and biomineralize calcite have made this genus one of the most promising for biomineralization as a biotechnological tool for the construction industry and bioremediation (Phillips et al., 2013; Zhu and Dittrich, 2016). It was also possible to determine that there are different strains of the same bacterial species, which have distinctly evident characteristics.

Knorre and Krumbein (2000) described bacteria isolated from freshwater, marine, and hypersaline environments as being capable of precipitating carbonates. However, there are differences in the mineralogy of precipitates that depends on the species of halophilic bacteria involved (Ferrer et al., 1988; Rivadeneyra et al., 2006). Halophilic bacteria, such as *Halomonas* and *Salinivibrio*, have been reported to precipitate calcium, magnesium carbonates, and phosphates by bypassing the inhibitory effect of high Mg^2+^ ion concentrations in bacteria-mediate precipitation of CO${}_{3}^{2+}$ (Raz et al., 2000; Rivadeneyra et al., 2006). In addition, one of advantages of using halophilic bacteria is their low potential for pathogenicity (Stabnikov et al., 2013).

### Biomineralization Applications in Seawater

In recent years, freshwater consumption has reached critical levels in Northern Chile, as they have around the world. Industrial use places great pressure on this resource, especially in arid areas where there is a shortage of freshwater resources. Desalinated and non-desalinated seawater hold a real alternative for sustained industrial development in these regions. However, its use has disadvantages, mostly due to problems with chemical composition and high concentrations of calcium and magnesium ions, which have a negative effect on reverse osmosis plants and other industries, such as copper and molybdenum mining.

All bacteria with urease activity described in this study can precipitate calcium crystals from ions present in seawater in different percentages and times. In biomineralization tests in seawater, *Bacillus subtilis* and *Halomonas* sp. were able to biomineralize both calcium (96–97%) and magnesium (63–67%) ions over 14 days of testing. SEM–EDX and XRD analyses determined that both bacteria induce the formation of 9–33% halite (NaCl), 31–66% monohydrocalcite (CaCO_3_ × H_2_O), and 24–27% struvite (MgNH_4_PO_4_ × 6H_2_O). Additionally, *B.subtilis* induces the formation of 7% anhydrite (CaSO_4_).

These results suggest that bacterial ureolytic metabolism in culture media produces the necessary conditions to induce heterogeneous calcium carbonate crystallization (Whiffin et al., 2007; Stabnikov et al., 2013). Le Corre et al. (2009) and Pastor et al. (2008) described how calcium interferes with struvite formation, inhibiting the reaction by blocking active struvite growth sites and competing for orthophosphate to form calcium phosphate. Given that the highest percentage of calcium is removed from the solution in the first 3 days of culture (84%), this would permit the subsequent precipitation of magnesium through struvite formation. Struvite precipitation depends on different other factors, such as pH, the Mg^2+^:NH${}_{4}^{+}$:PO${}_{4}^{3-}$ molar ratio, concentrations of potentially interfering ions, and ionic strength (Desmidt et al., 2013). The ability to form struvite is not widely described in nature (Laiz et al., 2009). Rivadeneyra et al. (1983, 1992b) have described struvite precipitation by *Pseudomonas, Flavobacterium, Arthrobacter* (Rivadeneyra et al., 1983, 1992a,b), and *Chromohalobacter* species (Rivadeneyra et al., 2006). Also, Sánchez-Román et al. (2007) found that moderately halophilic gamma-proteobacteria, including *Halomonas* spp., *Salinivibrio costicola, Marinomonas communis*, and *Marinobacter hydrocarbonoclasticus*, precipitate struvite.

Additionally, several authors have found that an increase in pH, as a result of the metabolic activity of the bacteria, provides the necessary ions for the formation of minerals, e.g., NH${}_{4}^{+}$ and PO${}_{4}^{3-}$ for struvite or CO${}_{3}^{2-}$ for carbonates (Sánchez-Román et al., 2007; Silva-Castro et al., 2013). This clearly demonstrates that bacteria do not act simply as a nucleation site for biomineralization but, rather, act as active mediators in the process (Sánchez-Román et al., 2007; Silva-Castro et al., 2013).

According to the results determined in the biomineralization tests, the biomineralization of *B.subtilis* strain LN8B and *Halomonas* sp. grown in the seawater biomineralization test have a tendency to form calcium crystals. This is followed by the biomineralization of magnesium crystals. This could be because the Ca^2+^ ion is adsorbed more frequently than the Mg^2+^ ion on the negatively charged cellular envelope of the bacteria, which has a greater power for ionic selectivity (Rivadeneyra et al., 1998; Silva-Castro et al., 2013). Liquid media and high salt concentrations seem to favor the formation of monohydrocalcite. In this context, our results are consistent with those described by Rivadeneyra et al. (2004), who found that, in the cultures of *Halobacillus trueperi*, monohydrocalcite is precipitated only in liquid media with high salt concentrations. Our results are different from those previously described by Rivadeneyra et al. (1991), which indicated that the presence of magnesium ions may prevent the precipitation of calcium carbonate crystals. This is because the inhibitory effect of magnesium mostly affects non-halotolerant, rather than halotolerant, bacteria (Rivadeneyra et al., 2006; Delgado et al., 2008).

The use of free bacterial cells for the removal of Ca^2+^ and Mg^2+^ from SW may not be fully feasible due to the need for a further separation of the biomass suspended from SW or due to low cell viability when faced with mechanical changes (Khoo and Ting, 2001). However, one of the main advantages of immobilized enzymes or microorganisms is that they can be used in continuous flow reactors because of their importance in industrial applications (Moynihan et al., 1989). The results obtained with *B.subtilis* LN8B immobilized in spheres of PV-Al, including urea and without LB, demonstrated the removal of 100% of Ca^2+^ at 8 days of testing and the removal of 62% of Mg^2+^ at 15 days. These values are similar to those observed in the free cell assays (Figures 4, 7). Unlike in our study, it had only been known that the partially purified *S. pasteurii* (previously *Bacillus pasteurii*) urease was immobilized in polyurethane (PU) foam to compare the efficacy of calcite precipitation between the free and immobilized enzymes. The immobilized urease showed higher Km and lower V_max_ values, which were reflected by slower overall calcite precipitation (Bachmeier et al., 2002). According to Bachmeier et al. (2002), the increase in pH is due to the presence of ammonium ions in the medium. This, in turn, is a consequence of the hydrolysis of urea accelerating the biomineralization rate of calcite, even with the immobilized enzyme. The biomineralization assays with *B.subtilis* LN8B immobilized in PV-Al spheres show that the interaction between ions and the surface of the bacteria would not be essential for the formation of calcite, in our case. However, it was not feasible to evaluate the distribution of the bacteria in the spheres, which does not allow for ruling out a feasible interaction. On the other hand, PV-Al is a water-soluble polymer that acts as a colloid mainly used for the active release of detergents and agrichemicals. This allows the interaction between the bacteria and the surrounding medium.

## Conclusions

Calcium biomineralization has only been tested through urea hydrolysis in freshwater and biomineralization in seawater from the ions present. These tests only allow us to improve water quality with respect to these ions, but biomineralization has not been tested in other types of water with other types of ions or contaminants, such as heavy metals or radionuclides.

Through the exploration of the bacterial capacity to precipitate calcium and magnesium ions from SW, it has been demonstrated that *B.subtilis* strain LN8B and *Halomonas* sp. strain LN12ABN have the biomineralization capability to precipitate these ions. The bacterial metabolism of both bacteria induces the formation of calcium biominerals (such as monohydrocalcite and anhydrite) and magnesium biominerals (such as struvite) from the ions present in SW. Additionally, *Halomonas* sp. LN12ABN formed 9% halite and *B.subtilis* formed 33%.

Despite the success in the search for bacteria able to precipitate calcium and magnesium from SW, the components needed to optimize the precipitation of these ions, the design of a bioreactor to pretreat the SW, and the means of immobilizing bacteria in a matrix that increases their survival and efficiency in ion removal are currently still under study. However, the positive results of these preliminary experiments certainly justify further development of the process.

Finally, this study reinforces the application of biomineralization mediated by bacteria as a biotechnological tool to improve the quality of SW for industrial processes and, alternatively, as reverse osmosis pretreatment.

## Author Contributions

DA performed all the experiments and the analysis of the results. CM performed all the statistical analyzes and contributed to the writing of the manuscript. DA also participated in writing this article. LAC participated in writing this article. MR participated in the design of the trials, analysis of results, and in writing this article.

### Conflict of Interest Statement

The authors declare that the research was conducted in the absence of any commercial or financial relationships that could be construed as a potential conflict of interest.

## Abbreviations

SEM–EDX: scanning electron microscopy with energy dispersive X-ray
XRD: X-ray diffraction.
